# Migraine-Asthma Comorbidity and Risk of Hypertensive Disorders of Pregnancy

**DOI:** 10.1155/2012/858097

**Published:** 2012-08-13

**Authors:** Stefanie Czerwinski, Jolana Gollero, Chunfang Qiu, Tanya K. Sorensen, Michelle A. Williams

**Affiliations:** ^1^Multidisciplinary International Research Training Program, University of Washington, Seattle, WA 98195, USA; ^2^Center for Perinatal Studies, Swedish Medical Center, Seattle, WA 98104, USA; ^3^Department of Epidemiology, Harvard School of Public Health, Boston, MA 02115, USA

## Abstract

*Background*. To evaluate the association of migraine and asthma and to estimate the risk of hypertensive disorders of pregnancy in relation to maternal comorbid migraine and asthma. *Methods*. Reproductive age women (*N* = 3.731) were interviewed during early pregnancy. At the time of interview, we ascertained participants' migraine and asthma status. From medical records, we collected information to allow the diagnosis of pregnancy-induced hypertension (PIH) and preeclampsia. Odds ratios (ORs) and 95% confidence intervals (CIs) were estimated using logistic regression procedures. *Results*. After adjusting for confounders, migraineurs had 1.38-fold increased odds of asthma as compared with nonmigraineurs (95% CI 1.09–1.38). The odds of hypertensive disorders of pregnancy were highest among women with comorbid migraine-asthma. The ORs for PIH preeclampsia and the two disorders combined were 2.53 (95% CI 1.39–4.61), 3.53 (95% CI 1.51–8.24), and 2.64 (95% CI 1.56–4.47), respectively, for women with comorbid migraine-asthma as compared with those who had neither disorder. *Conclusion*. These findings confirm prior reports and extend the literature by documenting particularly high odds of pregnancy-induced hypertension and preeclampsia among women with comorbid migraine-asthma. Increased knowledge about the prevalence and sequelae of comorbidities during pregnancy may lead to improved symptom management and perinatal outcomes.

## 1. Introduction

Migraine, a recurrent neurovascular headache disorder, is characterized by episodes of severe throbbing, pulsatile headache associated with nausea, vomiting, photophobia, phonophobia, and aversion to physical activity [1, 2]. The prevalence of migraine rises from 4% before puberty to a peak of 25% in women during their childbearing years, with a decrease after menopause [3]. An estimated 35 million US adults (18% of women and 6% of men) are thought to suffer from migraine [4]. Asthma, an inflammatory disease of the lower respiratory tract manifests as intermittent constriction of the bronchial airways [5]. Asthma attacks can cause a multitude of symptoms ranging in severity from mild to life-threatening. These symptoms include wheezing, breathlessness, chest tightness, and coughing. Approximately 8% of the US population reported that they currently have asthma [6]. Both conditions are known to negatively affect the overall quality of life by limiting physical activity [7, 8], and adversely impacting sleep [9]. Most population-based [10, 11] and clinical [12, 13], but not all [14, 15], studies have documented associations between migraine and asthma. As early as 1977, some investigators elected to describe asthma as “*pulmonary migraine*” [16] or “*acephalgic migraine*” [17].

Both migraine and asthma are prevalent among pregnant women; both conditions are known to be associated with adverse pregnancy outcomes [18–22]. For example, investigators have reported increased risks of preeclampsia among migraineurs [20, 22–24], and others have noted increased risk of the condition among asthmatics [25–27]. The high prevalence of migraine and asthma together with the potential for cooccurrence of these disorders is a concern because a diagnosis of one should lead to increased vigilance for screening and treating the other. There are, however, no published reports on the cooccurrence of migraine and asthma among pregnant women. We, therefore, examined the relationship between migraine and asthma in a well-characterized cohort of pregnant women who were interviewed during early pregnancy. We also investigated the association of comorbid migraine-asthma with the incidence of pregnancy-induced hypertension and preeclampsia. We hypothesize that women with comorbid migraine-asthma would have the highest risk of these hypertensive disorders of pregnancy.

## 2. Methods

### 2.1. Study Design and Setting

 We analyzed data from the Omega Study, a prospective cohort study designed to examine risk factors of adverse pregnancy outcomes including preeclampsia. Participants were recruited from women attending prenatal care clinics affiliated with Swedish Medical Center and Tacoma General Hospital in Seattle and Tacoma, WA, USA. Recruitment began in December 1996 [28]. The study protocol was approved by the Institutional Review Boards of Swedish Medical Center and Tacoma General Hospital. All participants provided informed consent.

Eligible women were those who began prenatal care before 20 weeks of gestation, spoke and read English, were ≥18 years of age, and planned to carry the pregnancy to term and deliver at either of the two hospitals. During early pregnancy, participants were asked to complete a structured interviewer administered questionnaire regarding sociodemographic characteristics, lifestyle habits, and medical and reproductive histories. Pregnancy outcome information was abstracted from hospital and clinic medical records.

### 2.2. Analytical Population

The analytical study population was derived from participants enrolled in the Omega Study between 1996 and 2008. During this period, 5,063 eligible women were approached and 4,000 (approximately 79%) agreed to participate. Women with incomplete information concerning prior history of migraine (*n* = 267) or asthma diagnosis (*n* = 2) were excluded from this analysis. A cohort of 3,731 women remained for analysis.

### 2.3. Data Collection

From structured questionnaire and medical records, we obtained information of covariates including maternal age, educational attainment, self-reported height, pre-pregnancy weight, reproductive and medical histories, and medical histories of first-degree family members. We also collected information on maternal smoking during pregnancy. Pre-pregnancy body mass index (BMI) was calculated as pre-pregnancy weight in kilograms divided by height in meters squared. Maternal history of migraine diagnosis was determined by response to the question *“Has a doctor ever told you that you have migraine headache?”* Similarly, maternal history of asthma diagnosis was determined by response to the question *“Has a doctor ever told you that you have asthma?”*

Maternal medical records were reviewed to collect detailed medical and clinical information. The diagnosis of pregnancy-induced hypertension (PIH) and preeclampsia (PE) was made using abstracted medical record data according to American College of Obstetricians and Gynecologists (ACOG) guidelines [29]. These guidelines defined preeclampsia as new onset hypertension with proteinuria in women who are beyond 20 weeks of gestation. Hypertension was defined as sustained blood pressure readings of ≥140/90 mmHg taken ≥6 hours apart. Proteinuria was defined as urine protein concentrations of 30 mg/dL on two or more random specimens collected at least 4 hours apart. Hereinafter, the term “pregnancy-induced hypertension (PIH)” is used to describe those women with hypertension without proteinuria. The term “preeclampsia” is used to describe those women with both pregnancy-induced hypertension and proteinuria.

### 2.4. Statistical Analyses

We compared the frequency distribution of sociodemographic, lifestyle, behavioral and medical history characteristics of participants according to whether or not they had received a physician diagnosis of migraine prior to the index pregnancy. We used unadjusted and multivariable-adjusted logistic regression models to calculate odds ratios (ORs) and 95% confidence intervals (CIs) of the association between migraine and asthma diagnosis. We also estimated unadjusted and multivariable adjusted odds ratios and 95% confidence intervals to evaluate the joint effect/comorbid effect of migraine and asthma history on risk of hypertensive disorders in the index pregnancy. For these analyses, we classified women by the joint distribution of prior history of migraine diagnosis (no versus yes) and prior history of asthma (no versus yes). The joint distribution of the two disorders resulted in the following four categories: no migraine and no asthma; history of migraine only; history of asthma only; comorbid migraine and asthma. Rothman and Greenland [30] have previously described this analytical approach. Polynomial logistic regression procedure was used to derive OR estimates for the ordinal outcomes (PIH and preeclampsia). We assessed confounding by entering covariates into the logistic regression model one at a time and adjusted ORs were compared to unadjusted ORs. Final logistic regression models included covariates that altered unadjusted ORs by 10%, as well as maternal age, race/ethnicity, parity, marital status, cigarette smoking during pregnancy, history of chronic hypertension or diabetes, pre-pregnancy body mass index, and multifetal pregnancy. We repeated analyses after excluding gravidas with prior histories of chronic hypertension or gestational diabetes, as well as those with multifetal pregnancies. Findings from these sensitivity analyses were largely similar to results from our primary analyses. We present continuous variables as mean with standard deviation. All reported *P* values are 2-tailed with statistical significance set at 0.05.

## 3. Results

The sociodemographic, medical, and behavioral characteristics of study participants are summarized in Table 1. Approximately 18% of women in this cohort reported a history of migraine. Migraineurs and nonmigraineurs were similar with regards to maternal age, race/ethnicity, educational attainment, marital and smoking status. Migraineurs were less likely to be nulliparous and were more likely to have a family and personal history of chronic hypertension, as compared with nonmigraineurs. Moreover, migraineurs were more likely to be obese than nonmigraineurs.

The overall prevalence of asthma in this cohort was 13.1%. As shown in Table 2, migraineurs had a 1.44-fold increased odds of asthma as compared with nonmigraineurs (unadjusted OR = 1.44; 95% CI 1.14–1.81). After adjusting for maternal age, parity, marital status, history of chronic hypertension, and pre-pregnancy body mass index, the odds ratio was slightly attenuated, though the association remained statistically significant (adjusted OR = 1.38; 95% CI 1.09–1.74).

We next evaluated the risk of PIH, preeclampsia, and the two disorders combined (any hypertensive disorder of pregnancy) in relation to maternal migraine only, asthma only, and comorbid migraine-asthma status (Table 3). After adjusting for maternal age, race/ethnicity, parity, marital status, cigarette smoking, history of chronic hypertension and pre-pregnancy body mass index, women with comorbid migraine-asthma had a 2.53-fold increased odds of pregnancy-induced hypertension (95% CI 1.39–4.61) as compared with those women with no history of migraine or asthma (i.e., the reference group). Further adjustment for multifetal pregnancies did not materially alter the observed association (adjusted OR = 2.55; 95% CI 1.40–4.63). There was no clear evidence of associations of isolated migraine (adjusted OR=1.20; 95% CI 0.84–1.71) or asthma only (adjusted OR = 1.04; 95% CI 0.66–1.63) with the occurrence of pregnancy-induced hypertension.

Women with a history of asthma only had a 1.89-fold increased odds of preeclampsia as compared with the referent group (95% CI 1.08–3.28). No similar association was observed for women with the diagnosis of migraine only (adjusted OR=0.83; 95% CI 0.46–1.50). Comorbid migraine-asthma was associated with a 3.53-fold increased odds of preeclampsia (adjusted OR = 3.53; 95% CI 1.51–8.24). Similar patterns of associations were observed when pregnancy-induced hypertension and preeclampsia were combined and evaluated as any hypertensive pregnancy disorder. We repeated the analysis summarized in Table 3 after excluding participants with multifetal pregnancy and prior histories of chronic hypertension and diabetes. Results from these sensitivity analyses are included in Table 4. For example, when we assessed the occurrence of hypertensive disorders of pregnancy among women with no history of pregestational diabetes, the proportions were 0.09, 0.13, 0.13, and 0.23 for those with no migraine and no asthma, those with a history of migraine only, those with a history of asthma only, and those comorbid migraine and asthma, respectively. Overall, results from our sensitivity analyses are similar to those results reported in Table 3. 

## 4. Discussion

Women with a history of migraine had statistically significantly higher odds of asthma when compared with nonmigraineurs (adjusted OR = 1.38; 95% CI 1.09–1.38). Additionally, the odds of hypertensive disorders of pregnancy were found to be highest among women with comorbid migraine-asthma. The ORs for PIH and preeclampsia and the two disorders combined were 2.53 (95% CI 1.39–4.61), 3.53 (95% CI 1.51–8.24), and 2.64 (95% CI 1.56–4.47), respectively, for women with comorbid migraine-asthma when compared with those women who had neither disorder. To the best of our knowledge, this is the first study examining the relationship between migraine and asthma in pregnant women. Although there are no published findings for this relationship specific to pregnant woman, our findings are consistent with population-based [10, 11] and clinic or hospital-based [12, 13] cross-sectional studies reporting associations of migraine, headache, and asthma.

Aamodt et al. [31] reported a positive association of both migraine and other nonmigrainous headache with asthma among 51,383 Norwegian participants of the Head-HUNT study. After adjusting for confounding by age, gender, education, and smoking, the authors noted that the odds of asthma was increased 1.5-fold (adjusted OR = 1.5; 95% CI 1.3–1.7) among migraineurs, as compared with nonmigraineurs. Non-migrainous headache was also associated with a 1.5-fold increased risk of asthma in this population (adjusted OR = 1.5; 95% CI 1.3–1.6). In a case-control study of 64,678 pairs of migraineurs and nonmigraineurs patients, matched on general practice, gender, and age, Davey et al. [13] observed increased relative risk of asthma (RR = 1.59; 95% CI 1.54–1.65) among migraineurs. Similarly, Lateef et al. [11] in their recent study of 10,198 children (age 4–18 years), who participated in the National Health and Nutrition Examination Surveys (NHANES), reported that children with headaches had a 1.7-fold increased odds of asthma (OR = 1.67; 95% CI 1.35–2.06) as compared with those children without headache individuals with headache as compared to those without. Von Behren et al. [32] also identified a positive association between self-reported lifetime prevalence of migraine and asthma. Using data from the 1998 California Behavioral Risk Factor Surveillance System (BRFSS), the authors noted that the lifetime prevalence of asthma was 13.9%. They also reported that women with a history of migraine had higher odds of a lifetime history of asthma than those without a history of migraine (prevalence ratio (PR) = 1.58; 95% CI 1.26–2.00). No such association was observed among men (PR = 1.12; 95% CI 0.69–1.82).

The pathogenesis for comorbid migraine and asthma remains unknown. However, investigators have speculated that migraine may share some common etiologic factors (e.g., genetic, biochemical, or environmental factors) with asthma and that a causal relationship exists between the two disorders [33, 34]. For example, alterations in inflammatory and vasoactive mediators including complement and immunoglobulins, histamine, cytokines, and nitric oxide or derangements in arachidonic acid metabolism [35] may underlie the development of migraine and other headaches among asthmatics [34, 36–38]. Investigators have postulated that histamine may be an important trigger of a cascade of neurogenic events that may lead to migraine or other primary headache disorders by means of CNS vasodilation or by stimulation of sensory nerve fibers [34]. Alternatively, migraine and headache disorders may be a secondary manifestation of allergies, or a sequelae of its treatment [34]. For instance, use of beta-blockers, salicylates, and nonsteroidal anti-inflammatory medications to treat migraine may trigger asthma [35, 39] in some patients. Longitudinal studies are needed to clarify the temporal relation of the symptoms and diagnoses of each disorder and to empirically evaluate these intriguing mechanistic hypotheses.

Migraine and asthma are prevalent medical conditions among reproductive aged women, and both conditions are known to be associated with adverse perinatal outcomes [18–22]. Marcoux and colleagues, in their case-control study of Canadian women [40], noted that those with a history of migraines had a 2.4-fold increased risk of preeclampsia as compared with those who did not have migraines (OR = 2.4; 95% CI 1.4–4.2). Adeney et al. [22] in their case-control study of American women residing in the Pacific Northwest region of the US reported a positive association between migraine history and preeclampsia risk. The authors noted that women with a history of physician-diagnosed migraine had a 1.8-fold (OR = 1.8; 95% CI, 1.1–2.7) increased risk of preeclampsia compared with women not having such a medical history. Similarly, Sanchez et al. [23] in their case-control study of Peruvian women reported that migraineurs had a 4.0-fold increased risk of preeclampsia (OR = 4.0; 95% CI 1.9–8.2) compared with nonmigraineurs. Additionally, Facchinetti et al. [24] in their prospective cohort study 702 normotensive Italian women with singleton pregnancies reported that migraine, classified using the International Headache Society diagnostic criteria, was associated with incident hypertensive disorders in pregnancy (OR = 2.85; 95% CI 1.40–5.81).

A substantial literature also suggests increased risks of hypertensive disorders of pregnancy among pregnant asthmatics [19, 25–27]. Liu et al. [26] in their retrospective study of 2,193 Canadian mothers with asthma and 8772 nonasthmatic mother noted preeclampsia risks among asthmatics (unadjusted OR = 1.80; 95% CI 1.36–2.39). Similar associations were reported by Demissie and colleagues (25) who noted that asthmatics in New Jersey had a 2.18-fold increased risk of preeclampsia as compared with non-asthmatics (OR = 2.18; 95% CI 1.68–2.83). In 2006, Rudra et al. [27] in a case-control study of 286 preeclampsia cases and 470 normotensive controls in Seattle, WA, USA, reported that women experiencing asthma symptoms during pregnancy were more likely than pregnant nonasthmatics to have preeclampsia (OR = 2.20; 95% CI 0.79–6.10), and that those with long-term pre-pregnancy asthma and symptoms during pregnancy were at particularly increased preeclampsia risk (OR = 9.09; 95% CI 1.02–81.6). Collectively, these studies and those from our present study suggest that pregnant women with migraine and/or asthma are high-risk patients that may benefit from particular attention paid to the management of these disorders during pregnancy [19, 21].

Several limitations of our study merit discussion and consideration. First, maternal migraine and asthma status was based on self-reports made during interviews and on medical records review. Although the questions we used to ascertain maternal migraine and asthma status have been widely used in National Health and Nutrition Examination Surveys (NHANES) and other large epidemiological studies, [11, 32] and investigators have documented good agreement between chronic disease classification based on self-reports with information derived from medical records review [41], we cannot exclude the possibility of that migraine and asthma status was underreported in our study. Studies that systematically use screening and confirmatory diagnostic evaluations will attenuate greatly concerns about misclassification of maternal migraine and asthma diagnoses in epidemiological studies. Second, although we adjusted for several potential confounders, we cannot exclude the possibility of residual confounding due to misclassification of adjusted variables (e.g., maternal pre-pregnancy body mass index) or confounding by other unmeasured variables (e.g., the severity and frequency of emergency asthmatic episodes and medications used during pregnancy). Lastly, the generalizability of our study may also be limited as our cohort was primarily comprised of non-Hispanic White and well-educated women. The concordance of our results with those from other studies that have included racially, ethnically, and geographically diverse populations, however, serves to attenuate these concerns.

In summary, we found associations of migraine and asthma in a cohort of pregnant women. This observed comorbidity was associated with markedly elevated odds of pregnancy-induced hypertension and preeclampsia. If confirmed in other pregnancy cohorts, observed comorbidity of migraine and asthma along with observed odds of adverse pregnancy outcomes among gravidas with comorbid migraine-asthma should further motivate increased vigilance in clinical evaluation and management of obstetric patients with these disorders [19, 21]. Additionally, future studies that seek to evaluate mechanistic hypotheses that may underlie the observed comorbidity and its associations with hypertensive disorders of pregnancy may yield new generalizable knowledge that could potentially lead to improvements in maternal and fetal outcomes.

## Figures and Tables

**Table 1:** Sociodemographic and other characteristics of the cohort, Seattle and Tacoma, WA, USA, 1996–2008.

	Entire Cohort	Physician-Diagnosed Migraine		
Characteristics	(*N* = 3,731)	Yes (*N* = 670)	No (*N* = 3,061)	*P* Value
	%	%	%	
Maternal age (years)	32.6 ± 4.5*	32.5 ± 4.6	32.7 ± 4.5	0.27
<20	0.6	0.5	0.6	
20–29	22.2	25.8	21.4	
30–34	43.6	40.6	44.3	0.14
35–39	27.0	26.3	27.1	
≥40	6.6	6.9	6.5	
Maternal race/ethnicity				
Non-Hispanic white	86.4	88.7	85.9	
African American	1.9	1.8	1.9	0.15
Other	11.7	9.5	12.2	
Annual household income ($)				
<30,000	3.4	3.0	3.5	
30,000–69,999	19.8	20.8	19.6	0.79
≥70,000	73.4	72.7	73.6	
Missing	3.3	3.6	3.3	
Nulliparous	62.5	58.2	63.4	0.01
Education ≤ high school	4.1	4.6	3.9	0.40
Unmarried	9.0	10.5	8.7	0.14
History of diabetes mellitus	1.3	1.3	1.3	0.89
History of chronic hypertension	4.7	8.2	4.0	<0.001
Family history of diabetes mellitus	14.7	16.4	14.4	0.18
Family history of hypertension	50.0	53.1	49.3	0.07
Not employed during pregnancy	18.6	17.6	18.8	0.49
Smoked during pregnancy	5.8	6.1	5.7	0.71
No prenatal vitamin use	2.7	2.8	2.7	0.78
No exercise during pregnancy	13.1	12.5	13.2	0.66
Pre-pregnancy body mass index (kg/m^2^)	23.6 ± 5.0*	24.5 ± 6.1	23.4 ± 4.7	<0.001
<18.5	4.3	3.9	4.3	
18.5–24.9	70.3	67.0	71.0	0.003
25–29.9	16.4	16.4	16.4	
≥30	9.0	12.7	8.2	
Multiple births	3.2	2.5	3.3	0.29
Gestational age at delivery (weeks)	38.4 ± 3.5*	38.3 ± 3.4	38.4 ± 3.5	0.54
Infant birth weight (gram)	3408 ± 604*	3406 ± 589	3409 ± 608	0.90

^∗^Mean ± standard deviation (SD).

**Table 2:** Unadjusted and adjusted odds ratios (ORs) and 95% confidence intervals of asthma and migraine comorbidity, Seattle and Tacoma, WA, USA, 1996–2008.

	Physician-Diagnosed Migraine			
	Yes (*N* = 670)	No (*N* = 3,061)	Unadjusted OR (95% CI)	^ ∗^ Adjusted OR (95% CI)
	*n* (%)	*n* (%)		
Physician-diagnosed asthma				
No	558 (83.3)	2686 (87.7)	1.00 (reference)	1.00 (reference)
Yes	112 (16.7)	375 (12.3)	1.44 (1.14–1.81)	1.38 (1.09–1.74)

^∗^ Adjusted for maternal age, parity, marital status, history of chronic hypertension, and pre-pregnancy body mass index.

**Table 3:** Unadjusted and adjusted odds ratios (ORs) and 95% confidence intervals (CIs) of hypertensive disorders of pregnancy in relation to maternal migraine and asthma status, Seattle and Tacoma, WA, USA, 1996–2008.

Outcomes	No migraine/no asthma	Migraine only	Asthma only	Comorbid Migraine-Asthma
Pregnancy-induced hypertension (PIH)				
No, *n*	2404	479	326	82
Yes, *n*	167	56	27	17
Unadjusted OR (95% CI)	1.00 (reference)	1.68 (1.22–2.31)	1.19 (0.78–1.82)	2.98 (1.73–5.15)
^ 1^Adjusted OR (95% CI)	1.00 (reference)	1.20 (0.84–1.71)	1.04 (0.66–1.63)	2.53 (1.39–4.61)
^ 2^Adjusted OR (95% CI)	1.00 (reference)	1.21 (0.85–1.74)	1.03 (0.66–1.62)	2.55 (1.40–4.63)

Preeclampsia (PE)				
No, *n*	2404	479	326	82
Yes, *n*	72	19	20	8
Unadjusted OR (95% CI)	1.00 (reference)	1.32 (0.79–2.22)	2.05 (1.23–3.41)	3.26 (1.52–6.99)
^ 1^Adjusted OR (95% CI)	1.00 (reference)	0.83 (0.46–1.50)	1.89 (1.08–3.28)	3.53 (1.51–8.24)
^ 2^Adjusted OR (95% CI)	1.00 (reference)	0.89 (0.49–1.63)	2.00 (1.14–3.49)	3.77 (1.61–8.82)

PIH or PE				
No, *n*	2404	479	326	82
Yes, *n*	239	75	47	25
Unadjusted OR (95% CI)	1.00 (reference)	1.58 (1.19–2.08)	1.45 (1.04–2.02)	3.07 (1.92–4.89)
^ 1^Adjusted OR (95% CI)	1.00 (reference)	1.13 (0.82–1.55)	1.27 (0.88–1.83)	2.64 (1.56–4.47)
^ 2^Adjusted OR (95% CI)	1.00 (reference)	1.15 (0.84–1.59)	1.28 (0.88–1.84)	2.69 (1.59–4.56)

Note: Subjects with unknown hypertensive disorder status were excluded from this analysis.

^1^Adjusted for maternal age, race/ethnicity, parity, marital status, cigarette smoking status, history of chronic hypertension, and pre-pregnancy body mass index.

^2^Adjusted for maternal age, race/ethnicity, parity, marital status, cigarette smoking status, history of chronic hypertension, pre-pregnancy body mass index, and multifetal pregnancy.

**Table 4:** Unadjusted and adjusted odds ratios (ORs) and 95% confidence intervals (CIs) of hypertensive disorders of pregnancy in relation to maternal migraine and asthma status, Seattle and Tacoma, WA, USA, 1996–2008.

	No Migraine/No Asthma	Migraine Only	Asthma Only	Comorbid Migraine-Asthma
Pregnancy-induced hypertension (PIH)				
No, *n*	2267	448	308	78
Yes, *n*	126	35	18	13
Unadjusted OR (95% CI)	1.00 (reference)	1.41 (0.95–2.07)	1.05 (0.63–1.75)	3.00 (1.62–5.54)
^ 1^Adjusted OR (95% CI)	1.00 (reference)	1.21 (0.81–1.81)	0.98 (0.58–1.65)	2.44 (1.27–4.69)

Preeclampsia (PE)				
No, *n*	2267	448	308	78
Yes, *n*	42	7	11	6
Unadjusted OR (95% CI)	1.00 (reference)	0.84 (0.38–1.89)	1.93 (0.98–3.78)	4.15 (1.71–10.06)
^ 1^Adjusted OR (95% CI)	1.00 (reference)	0.70 (0.30–1.63)	1.82 (0.91–3.61)	4.11 (1.65–10.19)

PIH or PE				
No, *n*	2267	448	308	78
Yes, *n*	168	42	29	19
Unadjusted OR (95% CI)	1.00 (reference)	1.27 (0.89–1.80)	1.27 (0.84–1.92)	3.29 (1.94–5.56)
^ 1^Adjusted OR (95% CI)	1.00 (reference)	1.11 (0.77–1.60)	1.19 (0.78–1.82)	2.69 (1.53–4.74)

Note: Subjects with history of chronic hypertension or preexisting diabetes mellitus or multifetal pregnancy were excluded from this analysis.

^1^Adjusted for maternal age, race/ethnicity, parity, marital status, cigarette smoking status, and pre-pregnancy body mass.

## References

[1] Katsnelson MJ, Peterlin BL, Rosso AL, Alexander GM, Erwin KL (2009). Self-reported vs measured body mass indices in migraineurs: research submission. *Headache*.

[2] Machado RB, Pereira AP, Coelho GP, Neri L, Martins L, Luminoso D (2010). Epidemiological and clinical aspects of migraine in users of combined oral contraceptives. *Contraception*.

[3] Lipton RB, Bigal ME, Diamond M, Freitag F, Reed ML, Stewart WF (2007). Migraine prevalence, disease burden, and the need for preventive therapy. *Neurology*.

[4] Ford ES, Li C, Pearson WS, Zhao G, Strine TW, Mokdad AH (2008). Body mass index and headaches: findings from a national sample of US adults. *Cephalalgia*.

[5] Murphy DM, O’Byrne PM (2010). Recent advances in the pathophysiology of asthma. *Chest*.

[6] (NCHS) NCfHS Health data interactive. http://www.cdc.gov/nchs/hdi.htm.

[7] Terwindt GM, Ferrari MD, Tijhuis M, Groenen SMA, Picavet HSJ, Launer LJ (2000). The impact of migraine on quality of life in the general population: the GEM study. *Neurology*.

[8] Varkey E, Hagen K, Zwart JA, Linde M (2008). Physical activity and headache: results from the Nord-Trøndelag Health Study (HUNT). *Cephalalgia*.

[9] Parish JM (2009). Sleep-related problems in common medical conditions. *Chest*.

[10] Ziegler DK, Hassanein RS, Couch JR (1977). Characteristics of life headache histories in a nonclinic population. *Neurology*.

[11] Lateef TM, Merikangas KR, He J (2009). Headache in a national sample of American children: prevalence and comorbidity. *Journal of Child Neurology*.

[12] Gürkan F, Ece A, Haspolat K, Dikici B (2000). Parental history of migraine and bronchial asthma in children. *Allergologia et Immunopathologia*.

[13] Davey G, Sedgwick P, Maier W, Visick G, Strachan DP, Anderson HR (2002). Association between migraine and asthma: matched case-control study. *British Journal of General Practice*.

[14] Lance JW, Anthony M (1966). Some clinical aspects of migraine. A prospective survey of 500 patients. *Archives of Neurology*.

[15] Schele R, Ahlborg B, Ekbom K (1978). Physical characteristics and allergic history in young men with migraine and other headaches. *Headache*.

[16] Tucker GF (1977). Pulmonary migraine. *Annals of Otology, Rhinology and Laryngology*.

[17] Hayashi T (1988). Asthma and migraine—is asthma a part of acephalgic migraine? A hypothesis. *Annals of Allergy*.

[18] Sorensen TK, Dempsey JC, Xiao R, Frederick IO, Luthy DA, Williams MA (2003). Maternal asthma and risk of preterm delivery. *Annals of Epidemiology*.

[19] Dombrowski MP (2006). Asthma and pregnancy. *Obstetrics & Gynecology*.

[20] Adeney KL, Williams MA (2006). Migraine headaches and preeclampsia: an epidemiologic review. *Headache*.

[21] Allais G, Gabellari IC, Borgogno P, De Lorenzo C, Benedetto C (2010). The risks of women with migraine during pregnancy. *Neurological Sciences*.

[22] Adeney KL, Williams MA, Miller RS, Frederick IO, Sorensen TK, Luthy DA (2005). Risk of preeclampsia in relation to maternal history of migraine headaches. *Journal of Maternal-Fetal and Neonatal Medicine*.

[23] Sanchez SE, Qiu C, Williams MA, Lam N, Sorensen TK (2008). Headaches and migraines are associated with an increased risk of preeclampsia in Peruvian women. *American Journal of Hypertension*.

[24] Facchinetti F, Allais G, Nappi RE (2009). Migraine is a risk factor for hypertensive disorders in pregnancy: a prospective cohort study. *Cephalalgia*.

[25] Demissie K, Breckenridge MB, Rhoads GG (1998). Infant and maternal outcomes in the pregnancies of asthmatic women. *American Journal of Respiratory and Critical Care Medicine*.

[26] Liu S, Wen SW, Demissie K, Marcoux S, Kramer MS (2001). Maternal asthma and pregnancy outcomes: a retrospective cohort study. *American Journal of Obstetrics and Gynecology*.

[27] Rudra CB, Williams MA, Frederick IO, Luthy DA (2006). Maternal asthma and risk of preeclampsia: a case-control study. *Journal of Reproductive Medicine for the Obstetrician and Gynecologist*.

[28] Qiu C, Luthy DA, Zhang C, Walsh SW, Leisenring WM, Williams MA (2004). A prospective study of maternal serum C-reactive protein concentrations and risk of preeclampsia. *American Journal of Hypertension*.

[29] Roccella EJ (2000). Report of the National High Blood Pressure Education Program Working Group on High Blood Pressure in Pregnancy. *American Journal of Obstetrics and Gynecology*.

[30] Rothman KJ, Greenland S (1998). *Modern Epidemiology*.

[31] Aamodt AH, Stovner LJ, Langhammer A, Hagen K, Zwart JA (2007). Is headache related to asthma, hay fever, and chronic bronchitis? The Head-HUNT study. *Headache*.

[32] Von Behren J, Kreutzer R, Hernandez A (2002). Self-reported asthma prevalence in adults in California. *Journal of Asthma*.

[33] Breslau N, Merikangas K, Bowden CL (1994). Comorbidity of migraine and major affective disorders. *Neurology*.

[34] Ku M, Silverman B, Prifti N, Ying W, Persaud Y, Schneider A (2006). Prevalence of migraine headaches in patients with allergic rhinitis. *Annals of Allergy, Asthma and Immunology*.

[35] Grzelewska-Rzymowska I, Bogucki A, Szmidt M (1985). Migraine in aspirin-sensitive asthmatics. *Allergologia et Immunopathologia*.

[36] Kemper RHA, Meijler WJ, Korf J, Ter Horst GJ (2001). Migraine and function of the immune system: a meta-analysis of clinical literature published between 1966 and 1999. *Cephalalgia*.

[37] Levy D, Burstein R, Kainz V, Jakubowski M, Strassman AM (2007). Mast cell degranulation activates a pain pathway underlying migraine headache. *Pain*.

[38] Özge A, Özge C, Kaleagasi H, Yalin OO, Ünal O, Özgür ES (2006). Headache in patients with chronic obstructive pulmonary disease: effects of chronic hypoxaemia. *Journal of Headache and Pain*.

[39] Covar RA, MacOmber BA, Szefler SJ (2005). Medications as asthma triggers. *Immunology and Allergy Clinics of North America*.

[40] Marcoux S, Berube S, Brisson J, Fabia J (1992). History of migraine and risk of pregnancy-induced hypertension. *Epidemiology*.

[41] Edwards WS, Winn DM, Collins JG (1996). Evaluation of 2-week doctor visit reporting in the national health interview survey. *Vital and health statistics. Series 2*.

